# Invasive pneumococcal disease burden in hospitalized adults in Bogota, Colombia

**DOI:** 10.1186/s12879-021-06769-2

**Published:** 2021-10-12

**Authors:** Paula O. Narváez, Salome Gomez-Duque, Juan E. Alarcon, Paula C. Ramirez-Valbuena, Cristian C. Serrano-Mayorga, Julian Lozada-Arcinegas, Alirio Bastidas, Sandra Gómez, Hernan Vargas, Charles Feldman, Luis Felipe Reyes

**Affiliations:** 1 Universidad de la Sabana, Campus Puente del Común, KM 7.5 Autopista Norte de Bogotá, Chía, Colombia; 2 Grupo Laboratorio de Salud Pública de Bogotá, Secretaría de Salud de Bogotá, Bogotá, Colombia; 3 Department of Internal Medicine, Faculty of Health Sciences, University of the Witwatersrand, Johannesburg, South Africa; 4 Clínica Universidad de la Sabana, Chía, Colombia

**Keywords:** Invasive pneumococcal disease (IPD), *Streptococcus pneumoniae *(Spn), Prevalence, Serotype replacement disease, Mortality, Hospitalization, Intensive care unit admission, Pneumococcal serotype distribution

## Abstract

**Background:**

The incidence of invasive pneumococcal disease (IPD) varies depending on a number of factors, including vaccine uptake, in both children and adults, the geographic location, and local serotype prevalence. There are limited data about the burden of *Streptococcus pneumoniae* (*Spn*), serotype distribution, and clinical characteristics of adults hospitalized due to IPD in Colombia. The objectives of this study included assessment of *Spn* serotype distribution, clinical characteristics, mortality, ICU admission, and the need for mechanical ventilation.

**Methods:**

This was an observational, retrospective, a citywide study conducted between 2012 and 2019 in Bogotá, Colombia. We analyzed reported positive cases of IPD from 55 hospitals in a governmental pneumococcal surveillance program. Pneumococcal strains were isolated in each hospital and typified in a centralized laboratory. This is a descriptive study stratified by age and subtypes of IPD obtained through the analysis of medical records.

**Results:**

A total of 310 patients with IPD were included, of whom 45.5% were female. The leading cause of IPD was pneumonia (60%, 186/310), followed by meningitis. The most frequent serotypes isolated were 19A (13.87%, 43/310) and 3 (11.94%, 37/310). The overall hospital mortality rate was 30.3% (94/310). Moreover, 52.6% (163/310 patients) were admitted to the ICU, 45.5% (141/310) required invasive mechanical ventilation and 5.1% (16/310) non-invasive mechanical ventilation.

**Conclusion:**

Pneumococcal pneumonia is the most prevalent cause of IPD, with serotypes 19A and 3 being the leading cause of IPD in Colombian adults. Mortality due to IPD in adults continues to be very high.

**Supplementary Information:**

The online version contains supplementary material available at 10.1186/s12879-021-06769-2.

## Introduction

Lower respiratory tract infections are the leading cause of death in developing countries. Among respiratory tract infections, invasive pneumococcal disease (IPD) is an important cause of morbidity and mortality [1, 2]. According to the United States Active Bacterial Core Surveillance, in 2017, the incidence of IPD was 24 cases per 100,000 in age ≥ 65 years [3, 4]. In Latin America and the Caribbean, it is estimated that every year, IPD is responsible for up to 28,000 deaths, 182,000 hospitalizations, and 1.4 million outpatient consults [5–7]. Annually, the burden of CAP and associated invasive diseases represent annual costs exceeding $17 billion in the United States and more than €10 billion in Europe [8–10]. It is essential to highlight that after the widespread usage of pneumococcal vaccines in children, the adult population presents the highest incidence of IPD, and its mortality has remained steady even after the extensive usage of broad-spectrum antibiotics and better vaccination coverage in children [4, 5, 11, 12]. Thus, pneumococcal continues to be considered as a public health care problem [1, 4].

Currently, 100 serotypes of *Streptococcus pneumoniae* (*Spn*) have been described [13]. Each pneumococcal serotype has a different genetic and phenotypical composition [5]. Some *Spn* are more frequently associated with the development of IPD, depending on various factors that include vaccine uptake and geographic location. Importantly, pneumococcal vaccines are the most effective strategy to reduce IPD incidence [13]. There are two types of anti-pneumococcal vaccines: pneumococcal conjugated vaccines (PCV 7, PCV 10, and PCV13) and a pneumococcal polysaccharide vaccine (PPSV23). PCV13 includes purified capsular polysaccharide of 13 serotypes of *Spn* that frequently cause pneumococcal disease (1, 3, 4, 5, 6A, 6B, 7F, 9V, 14, 19A, 19F, 18C and 23F). This vaccine has polysaccharide antigens conjugated to a protein carrier to improve the immunogenicity [14]. The PPSV23 includes 23 serotypes of pneumococci polysaccharides, which stimulate the immune system to produce antibodies [15]. Unconjugated polysaccharide vaccines induce a T‐cell response, without the establishment of B‐cell memory while conjugated polysaccharide vaccines as well as inducing T-cell response also establish B-cell memory that ensures long- term immunization effect [16–19]. Different studies have shown that more than 80% of healthy adults who receive PPSV23 develop antibodies against the serotypes contained in the vaccine; the immune response usually occurs within 2 to 3 weeks after vaccination in adults. The Centers for Disease Control and Prevention (CDC) recommends one dose of PPSV23 in immunocompetent adults older than 65 years and in people 2 through 64 years old with certain medical conditions or in adults who smoke cigarettes. Likewise, CDC also recommends PCV13 in patients older than 65 years immunocompetent under a “shared clinical decision making [20]. Importantly, in 2009, Colombia began vaccination with the heptavalent vaccine. Later, in 2010, the PCV-10 was introduced, and its usage was determined as mandatory in 2011 in pediatric population. According to local authorities, anti-pneumococcal vaccination coverage from 2011 to 2017 was approximately 90% in children under one year of age. Although, PCV-13 andPPSV-23 are recommended in > 18 and older adults, it is not included as mandatory in the national immunization program in Colombia [20].

Since the introduction of the mandatory universal pneumococcal vaccination in children, IPD epidemiology has changed significantly worldwide, not only for children, but for adults [21, 22]. Currently, studies are describing yearly increases in the incidence of IPD due to several non-vaccine serotypes [23]. In Colombia, some studies have reported serotypes 1, 14, 3 and 19A as the most prevalent serotypes causing IPD in children [24, 25]. However, these findings were obtained from small studies of children admitted to few hospitals and before the widespread use of PCV in young children. However, there is a lack of epidemiological data regarding IPD serotype distribution, and clinical characteristics of adults hospitalized due to IPD in Colombia. Thus, this study was designed to fill that gap in the literature. We hypothesized that adult patients hospitalized due to IPD in Colombia would be infected, more frequently, with pneumococcal serotypes not included in the conjugate vaccines. Also, that pneumonia would make up the largest share of reported IPD, with higher mortality in older adults. The current study was undertaken with the objectives of assessing the *Spn* serotype distribution, clinical diagnosis, mortality, ICU admission, length of hospital stay, and need for mechanical ventilation in adults admitted due to IPD.

## Material and methods

This was a retrospective multicenter observational study of patients from 55 hospitals, including public and private institutions, in Bogotá, Colombia, that provide health care to about 8 million people. The study was undertaken between January 2012 and January 2019. All patients older than 18 years of age who were diagnosed with IPD were identified and those cases with a fully available clinical history, and positive *Spn* cultures were included in the study. Patients in whom clinical data were incomplete or other bacteria were isolated (i.e., co-infections) were excluded. The flow chart indicating the selection process is part of the supplemental information in Additional file 1: Fig. S1. The Translational Science in Infectious Diseases and Critical Care Medicine Research Group from the Universidad de La Sabana developed this study protocol in collaboration with The Public Health Secretary of Bogotá city.

We used the bacterial isolates reported to The Public Health Secretary under a *Spn* surveillance program. All pneumococcal isolates were confirmed and typed by the National Center of Microbiology (CNM) in a centralized laboratory following the Quellung reaction using polyclonal antisera [26]. This study was approved, and the use of informed consent was waived the Institutional Review Board of the Clinica Universidad de La Sabana (2020_MED-23221). The use of informed consent was waived because this was a retrospective study, without any intervention, and used only a chart review to obtain clinical data. All methods were performed in accordance with the relevant guidelines and local and international regulation.

### Study definitions

We used international and well-accepted definitions for each variable and clinical diagnosis. IPD was defined as an infection confirmed by the isolation of *Spn* from a normally sterile site (e.g*.,* blood, cerebrospinal fluid, and pleural, joint, peritoneal fluid and/or respiratory fluid other than sputum) [27]. CAP was defined according to the American Thoracic Society and Infectious Diseases Society of America (ATS/IDSA) guidelines as an acute infection of the pulmonary parenchyma acquired outside of the hospital setting evidenced by radiological findings (e.g.*,* chest radiograph, computed tomography or pulmonary ultrasound) compatible with alveolar infiltration and characteristic clinical presentation (e.g.*,* acute onset of cough, fever, tachypnea, altered mental status, diaphoresis, etc.) [28]. Pneumococcal meningitis was defined as the identification of *Spn* in blood cultures and/or CSF in association with the clinical syndrome of meningitis (i.e*.,* fever, headache, meningismus, altered mental status, seizures, confusion or vomiting) According to the IDSA guidelines [29].

To reduce the risk of bias, only confirmed cases of IPD according to international clinical guidelines were included and all the clinical records were evaluated by trained personnel. Additionally, all missing information was not analyzed or inferred, and all clinical charts were reviewed blinded to the isolated pneumococcal serotypes to determine clinical outcomes.

### Variables

Data on gender, age, comorbidities, chronic medication, vaccination, physiological admission variables, blood count, bilirubin levels, liver enzymes, arterial blood gases, medical intervention, and antibiotic treatment were recorded for each patient. The initial severity of the disease was assessed using the Sequential Organ Failure Assessment (SOFA), quick Sequential Organ Failure Assessment (qSOFA) [30], and Acute Physiology and Chronic Health Evaluation II (APACHE II) [31] (Indicating higher mortality rate with higher scores of both SOFA scores and APACHE II).

### Outcomes

During hospitalization, all-cause hospital mortality was the primary outcome. The need for ICU admission, need for invasive mechanical ventilation or non-invasive mechanical ventilation, length of hospital and ICU stay were recorded and considered as secondary outcomes.

### Data collection

Hospitals sent all isolated *Spn* strains to a centralized laboratory where each one was characterized. Clinical records were sent to the district health secretary of Bogotá city, and a retrospective review of all medical records was carried out to gather clinical information. Data was recorded onto an electronic case report form (eCRF) hosted in the servers of the health secretary of Bogota and then exported for statistical analysis.

### Statistical analysis

For the statistical analysis, descriptive analysis was performed with measures of central tendency and dispersion for the quantitative variables, and frequencies with percentages for the qualitative variables. Numerical data are presented as mean (standard deviation, SD) or median (interquartile ranges, IQR) according to normality distribution. The serotype distribution of *Spn* was broken down by clinical diagnosis and age (*Spn* distribution was grouped into four age groups: 18–35 years, 36–50 years, 51–64 years, and ≥ 65 years). Finally, mortality, ICU admission, need for invasive mechanical ventilation or non-invasive mechanical ventilation, and all-cause hospital mortality were calculated for the group as a whole as well as by diagnosis of IPD. Quantitative variables were contrasted by fisher, and continuos variables were analyzed according to data distribution (i.e.*,* normality) by t-test the Mann Witney. A p < 0.05 was considered statistically significant. All statistical analyses were performed using a statistical package SPSS 25 and Graph Pad Prism for MAC, licensed for the Universidad de La Sabana.

## Results

**Fig. 1:**
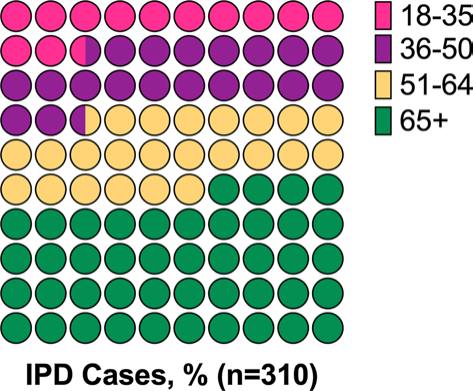
Distribution of age in adults who survived invasive pneumococcal disease (IPD) in Bogotá, Colombia. Stratified by age groups, 18–35, 36–50, 51–64 and ≥ 65 years old. Pink circles age ranges from 18 to 35, purple circles 36–50, yellow circles 51–64 and green circles age ≥ 65 years, being the most affected group by IPD

A total of 310 patients with invasive pneumococcal disease were included in the study; of whom 45.5% (141/310) were female. The mean (SD) age was 59 (± 18.7) years. Hypertension was the most frequent comorbidity in the patients (42.3% [131/310]), and (28.7% [89/310]) were smokers. The demographic information is documented in in Table 1 and Fig. 1 Patients were grouped according to their diagnosis. 60% (186/310) of the patients were diagnosed with CAP, followed by meningitis in 18.7% (58/310), and other diagnoses (mainly abdominal infections and primary bloodstream infections) in 21.3% (66/310) of the cases. Importantly, only 1.3% (4/310) had been previously immunized with PCV13 and 0.6% (2/310) with PPV 23 polysaccharide vaccine, and 1.3% (4/310) with influenza vaccine.

**Table 1:** Demographic and clinical characteristics of patients hospitalized due to invasive pneumococcal disease (IPD)

Patients characteristics	All patients (n = 310)	Pneumonia (n = 186)	Meningitis (n = 58)	Others (n = 66)
Gender and Age, n (%)				
Female	141 (45.5)	82 (44.1)	24 (41.4)	35 (53)
Age mean (SD)	58.7 (18.7)	62 (18.5)	50 (17.54)	56 (17.81)
Comorbidities, n (%)				
Smoking	89 (28.7)	64 (34.4)	13 (22.4)	12 (18.2)
Alcoholism	37 (11.9)	25 (13.4)	8 (13.8)	4 (6.1)
COPD	44 (14.2)	31 (16.7)	4 (6.9)	9 (13.6)
Diabetes	50 (16.1)	31 (16.7)	8 (13.8)	11 (16.7)
Hypertension	131 (42.3)	84 (45.2)	18 (31)	29 (43.9)
Heart failure	40 (12.9)	33 (17.7)	3 (5.2)	4 (6.1)
Chronic renal disease	36 (11.6)	27 (14.5)	4 (6.9)	5 (7.6)
Liver disease	17 (5.5)	10 (5.4)	2 (3.4)	5 (7.6)
Obesity	16 (5.2)	8 (4.3)	5 (8.6)	3 (4.5)
HIV	17 (5.5)	12 (6.5)	3 (5.2)	2 (3)
Coronary artery disease	26 (8.4)	20 (10.8)	1 (1.7)	5 (7.6)
Hospital admission in the prior month	47 (15.2)	38 (20.4)	0 (0)	9 (13.6)
Previous use of antibiotics	41 (13.2)	33 (17.7)	2 (3.4)	6 (9.1)
Dislipidemia	27 (8.7)	23 (12.4)	1 (1.7)	3 (4.5)
Atrial fibrillation	20 (6.5)	16 (8.6)	3 (5.2)	1 (1.5)
Autoimmune disease	38 (12.3)	24 (12.9)	7 (12.1)	7 (10.6)
Cancer	37 (11.9)	25 (13.4)	5 (8.6)	7 (10.6)
Epilepsy	5 (1.6)	3 (1.6)	1 (1.7)	1 (1.5)
Chronic medications, n (%)				
Antihypertensive drugs	131 (42.3)	90 (48.4)	16 (27.6)	25 (37.9)
Oral antidiabetic drugs	24 (7.7)	17 (9.1)	3 (5.2)	4 (6.1)
Insulins	17 (5.5)	11 (5.9)	3 (5.2)	3 (4.5)
Opioids	6 (1.9)	6 (3.2)	0 (0)	0 (0)
PPI	62 (20)	45 (24.2)	4 (6.9)	13 (19.7)
Inhaled drug therapy	25 (8.1)	17 (9.1)	1 (1.7)	7 (10.6)
Anticoagulant therapy	11 (3.5)	10 (5.4)	0 (0)	1 (1.5)
Statins	44 (14.2)	37 (19.9)	2 (3.4)	5 (7.6)
NSAID	16 (5.2)	9 (4.8)	2 (3.4)	5 (7.6)
Corticosteroid therapy	32 (10.3)	23 (12.4)	3 (5.2)	6 (9.1)
Antiepileptic drugs	6 (1.9)	3 (1.6)	2 (3.4)	1 (1.5)
Vaccination				
PPV23	2 (0.6)	2 (1.1)	0 (0)	0 (0)
PCV13	4 (1.3)	3 (1.6)	0 (0)	1 (1.5)
Influenza vaccine	4 (1.3)	3 (1.6)	0 (0)	1 (1.5)

SD, standard deviation; COPD, chronic obstructive pulmonary diseases; HIV, human immunodeficiency virus; PBI, proton pump inhibitor; NSAID, nonsteroidal anti-inflammatory drugs; PPV23, Pneumococcal polysaccharide vaccine; PVC13, Pneumococcal conjugate vaccine13

Physiological variables evaluated at admission were different between groups of patients. Regarding laboratory investigations on admission, we found that the C-reactive protein concentration (median (IQR)) was 36 mg/L (15.0–165.9 mg/L) in CAP, 47 mg/L (20.0–193.0) in meningitis, and 24 (13.05–38.90) in other diagnoses. The mean (SD) leukocyte counts were 12.44 × 10^9^/L (8.77) in CAP, 16.62 × 10^9^/L (8.61) in meningitis, and 12.54 × 10^9^/L (9.01) in other diseases; the complete list of physiological variables and laboratories are presented in (Table 2). When evaluating the severity of the disease in all hospitalized patients, the quick Sepsis Related Organ Failure Assessment (qSOFA), Sepsis Related Organ Failure Assessment (SOFA), and Acute Physiology and Chronic Health Evaluation (APACHE II) scores were calculated when possible. SOFA median (IQR) was 5 (3–8) in all patients with no significant difference among each group, while APACHE II varied between groups: median (IQR) score was 15 (11–20) in CAP, 13 (7–15) in meningitis, and 12 (9–20) in patients with other diagnoses (Table 2).

**Table 2:** Physiological variables of patients hospitalized due to Invasive Pneumococcal Disease (IPD)

Admission physiological variables. Mean (SD)+	All patients (n = 310)	Pneumonia (n = 186)	Meningitis (n = 58)	Others (n = 66)
Heart rate, bpm	97 (24)	99 (25)	97 (21)	94 (26)
Systolic blood pressure, mmHg	117 (26.3)	114 (26)	123 (26)	117 (24)
Diastolic blood pressure, mmHg	70 (16)	68 (17)	72 (14)	71 (16)
MAP, mmHg	84 (22)	81 (23)	88 (17.6)	85 (21)
Respiratory rate, rpm	21 (5)	21 (5)	20 (6)	20 (5)
Oximetry, %	88 (12)	87 (11)	92 (4)	87 (18)
Glasgow scale	14 (3)	14 (2.2)	12 (3)	14 (5)
Temperature, °C	36.92 (1.98)	37.05 (1.03)	37.26 (1.89)	36.24 (3.48)
Full blood count				
Leukocytes, ×10^9^/L	13.26 (8.91)	12.44 (8.77)	16.62 (8.61)	12.54 (9.01)
Neutrophils, 10^9^/L*	10 (5.2–16.4)	8.6 (4.2–15.85)	13.3 (8.22–21.45)	9.3 (5.09–15.00)
Hemoglobin, g/dL	12.9 (3.4)	12.52 (3.01)	14.13 (2.52)	13.02 (4.77)
Hematocrit, %	42.15 (40.25)	40.09 (29.96)	42.75 (7.82)	47.42 (71.13)
Platelets, ×10^3^*	201 (141–271)	209 (159–284.5)	188 (127.25–271.5)	179.5 (105.50–248.75)
Glucose, mg/dL*	119 (94–156)	119 (94–146.5)	131 (98.5–167)	114 (83.25–162.50)
Urea nitrogen, mg/dL*	21.9 (15–39.8)	23.8 (17–42)	17 (11–25.47)	20 (12.00–41.00)
Creatinine, mg/dL*	1.06 (0.7–1.7)	1.14 (0.79–1.81)	0.9 (0.7–1.2)	0.9 (0.70–2.12)
Sodium concentration, mmol/L	133 (20.43)	133 (19.18)	131.64 (24.74)	133.73 (19.97)
Potassium concentration, mmol/L*	3.9 (3.5–4.4)	3.9 (3.57–4.5)	3.7 (3.3–4.2)	4.1 (3.60–4.60)
Bilirubin levels				
Total bilirubin, mg/dL*	1.1 (0.6–2.2)	1.09 (0.58–1.9)	1.03 (0.52–2.23)	1.27 (0.63–3.34)
Direct bilirubin, mg/dL*	0.55 (0.3–1.14)	0.55 (0.29–1.12)	0.73 (0.2–1.15)	0.51 (0.30–1.36)
Indirect bilirubin, mg/dL*	0.5 (0.2–1.0)	0.45 (0.22–0.9)	0.47 (0.21–1.1)	0.57 (0.30–1.35)
Liver enzymes				
AST, UI/L*	39 (24–79)	36 (22.95–66.75)	28 (17–111)	47 (28.75–83.05)
ALT, UI/L*	36.2 (21–83.6)	35 (20–83.67)	37.4 (21–91)	43 (24.00–84.00)
Arterial blood gases				
pH*	7.42 (7.34–7.47)	7.4 (7.33–7.46)	7.44 (7.35–7.48)	7.43 (7.36–7.48)
PO2, mmHg	70.43 (36.16)	66.88 (38.71)	81 (35)	72.29 (22.33)
PCO2, mmHg	29.9 (12.59)	30.33 (10.82)	27.29 (8.18)	30.99 (20)
FiO2, %	30 (18)	30 (18)	33 (23)	25 (11)
HCO3, meq/L*	18 (14.47–20.65)	18 (14.5–21)	17 (14.2–19.5)	17 (14.62–20.27)
Lactate, mmol/L*	2.8 (1.9–4.82)	2.8 (1.9–5.3)	2.7 (1.77–3.82)	2.75 (1.62–4.57)
CRP, mg/L*	32 (15.29–133)	36 (15–165.9)	47 (20–193)	24 (13.05–38.90)
Procalcitonin	26 (56.79)	30.11 (67.29)	20.66 (36.81)	18.49 (36.23)
PT	15.63 (6.77)	14.86 (5.21)	16.84 (7.71)	16.21 (8.49)
PTT	34.49 (−19.51)	34 (9.44)	32.19 (11.64)	37.08 (33.96)

Clinical severity scores (IQR)	All patients	Pneumonia	Meningitis	Others
qSOFA	1 (0–2)	1 (0–2)	1 (0–2)	1 (1–2)
SOFA	5 (3–8)	5 (3–8)	5 (3–9)	5 (3–8)
APACHE II	13 (11–19)	15 (11–20)	13 (7–15)	12 (9–20)

SD, standard deviation; bpm, beats per minute; MAP, median arterial pressure; rpm, rate per minute; °C, Celsius degrees; ALT, Alanine aminotransferase, AST, Aspartate Aminotransferase; PO2, arterial oxygen pressure; PCO2, arterial carbon dioxide; FiO2, oxygen inspired fraction; HCO3, sodium bicarbonate; CRP, C-reactive protein; PT, prothrombin time; PTT, partial thromboplastin time; qSOFA, quick SOFA; SOFA, sequential organ failure assessment; APACHE II, acute physiology and chronic health evaluation II; PSI, Pneumonia severity index; + , mean ± SD, except where indicated; *, Median, (IQR)

Different investigations had been used to establish the etiology of the infection, including blood cultures, endotracheal fluid aspirate, cerebrospinal fluid (CSF), bronchoalveolar lavage, and pleural and ascitic fluid. Blood cultures were the most frequent sample used to identify the microorganism associated with infection. *Spn* was isolated in blood cultures in 88.1% (273/310) of the patients, followed by CSF (14.2% [44/310]), endotracheal fluid (8.4% [26/310]) and pleural fluid (6.5% [20/310]). *Spn* was identified in CSF in 70.7% (41/58) of the patients with meningitis (Additional file 1: Table S2). Regarding pneumococcal serotypes, the most prevalent *Spn* serotype identified as the cause of infection in the whole cohort was 19A in 13.9% (43/310) of the patients, 14% (27/186) were CAP patients, 17% (10/58) were patients with meningitis and 9% (6/66) had other presentations of IPD (Table 3). Table 3 shows the pneumococcal serotypes isolated.

**Table 3:** Serotype distribution of *Streptococcus pneumoniae* in patients hospitalized with invasive pneumococcal disease (IPD)

Serotype distribution (n (%))	All patients (n = 310)	Pneumonia (n = 186)	Meningitis (n = 58)	Others (n = 66)	P value
19A	43 (13.87)	27 (14.52)	10 (17.24)	6 (9.09)	0.40
3	37 (11.94)	27 (14.52)	4 (6.9)	6 (9.09)	0.13
14	18 (5.81)	11 (5.91)	2 (3.45)	5 (7.58)	0.61
15A	16 (5.16)	9 (4.84)	4 (6.9)	3 (4.55)	0.80
23A	16 (5.16)	6 (3.23)	4 (6.9)	6 (9.09)	0.14
6C	16 (5.16)	12 (6.45)	2 (3.45)	2 (3.03)	0.45
11A	14 (4.52)	9 (4.84)	4 (6.9)	1 (1.52)	0.33
9N	13 (4.19)	9 (4.84)	2 (3.45)	2 (3.03)	0.78
19F	8 (2.58)	2 (1.08)	2 (3.45)	4 (6.06)	0.08
6A	8 (2.58)	1 (0.54)	2 (3.45)	5 (7.58)	< 0.01
23B	6 (1.94)	4 (2.15)	1 (1.72)	1 (1.52)	0.94
15B	7 (2.26)	5 (2.69)	2 (3.45)	0 (0)	0.35
22F	7 (2.26)	4 (2.15)	0 (0)	3 (4.55)	0.23
8	7 (2.26)	5 (2.69)	1 (1.72)	1 (1.52)	0.82
1	6 (1.94)	5 (2.69)	0 (0)	1 (1.52)	0.41
10A	6 (1.94)	3 (1.61)	1 (1.72)	2 (3.03)	0.76
23F	6 (1.94)	3 (1.61)	2 (3.45)	1 (1.52)	0.65
7F	6 (1.94)	6 (3.23)	0 (0)	0 (0)	0.13
9V	6 (1.94)	4 (2.15)	1 (1.72)	1 (1.52)	0.94
16F	5 (1.61)	5 (2.69)	0 (0)	0 (0)	0.25
34	5 (1.61)	2 (1.08)	2 (3.45)	1 (1.52)	0.45
6B	5 (1.61)	3 (1.61)	1 (1.72)	1 (1.52)	0.99
13	4 (1.29)	3 (1.61)	0 (0)	1 (1.52)	0.62
18C	4 (1.29)	1 (0.54)	2 (3.45)	1 (1.52)	0.22
12B	3 (0.97)	1 (0.54)	1 (1.72)	1 (1.52)	0.21
20	3 (0.97)	2 (1.08)	0 (0)	1 (1.52)	0.67
28A	3 (0.97)	2 (1.08)	0 (0)	1 (1.52)	0.67
35B	3 (0.97)	1 (0.54)	1 (1.72)	1 (1.52)	0.63
35F	3 (0.97)	0 (0)	1 (1.72)	2 (3.03)	0.07
37	3 (0.97)	2 (1.08)	1 (1.72)	0 (0)	0.60
7C	3 (0.97)	3 (1.61)	0 (0)	0 (0)	0.36
12F	2 (0.65)	2 (1.08)	0 (0)	0 (0)	0.51
25F	2 (0.65)	1 (0.54)	1 (1.72)	0 (0)	0.46
31	2 (0.65)	1 (0.54)	0 (0)	1 (1.52)	0.55
35A	2 (0.65)	1 (0.54)	1 (1.72)	0 (0)	0.46
4	2 (0.65)	1 (0.54)	0 (0)	1 (1.52)	0.55
11D	1 (0.32)	0 (0)	0 (0)	1 (1.52)	0.15
15C	1 (0.32)	0 (0)	0 (0)	1 (1.52)	0.15
16	1 (0.32)	0 (0)	0 (0)	1 (1.52)	0.15
17F	1 (0.32)	0 (0)	1 (1.72)	0 (0)	0.11
18A	2 (0.65)	1 (0.54)	0 (0)	1 (1.52)	0.55
19C	1 (0.32)	1 (0.54)	0 (0)	0 (0)	0.71
24F	1 (0.32)	0 (0)	1 (1.72)	0 (0)	0.11
29	1 (0.32)	1 (0.54)	0 (0)	0 (0)	0.71
6D	1 (0.32)	0 (0)	1 (1.72)	0 (0)	0.11

N, number; %, percentage

**Fig. 2:**
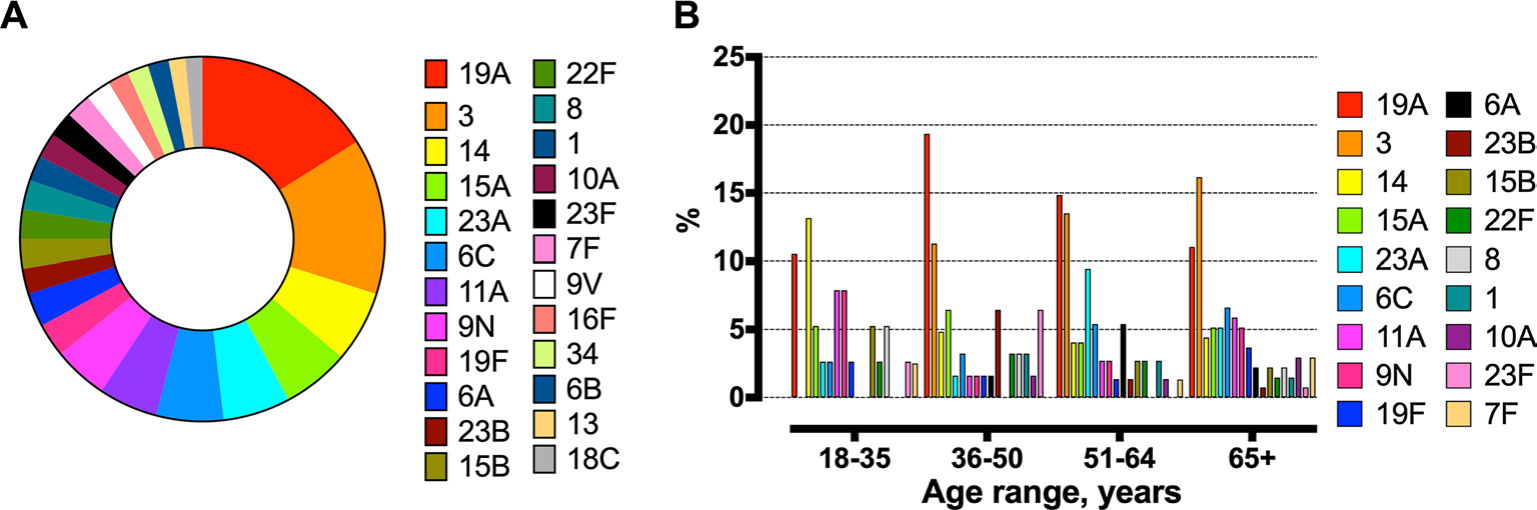
Overall pneumococcal serotype distribution (**A**) and distribution by age range (**B**)

The most prevalent Spn serotype was 19A followed by 3 and 14 (Table 3). Serotypes causing IPD in vaccinated patients with PCV13 were 3, 9N, 8 and 35B, while the ones vaccinated with PPV23 were infected by serotypes 9N and 8. The serotype distribution was grouped into four age groups, namely, 18–35 years, 36–50 years, 51–64 years, and ≥ 65 years. (Table 4) Serotype 19A was the most prevalent serotype among adult patients between 36 and 50 years (19% [12/62]) and in patients between 51 and 64 years (14.9% [11/74]). In contrast, serotype 3 caused IPD in 11.3% (7/62) of patients between 36 and 50 years and in 13.5% (10/74) of patients between 51 and 64 years. In patients ≥ 65 years old, serotype 3 (16.8% [22/136]) was the most prevalent, followed by serotype 19A (11% [15/136]). In the 18–35-year-old group, patients were mostly infected by serotype 14 (13.2% [5/38]). There were no significant differences in the serotype distribution between the four age groups. However, when the serotype distribution was stratified in two age groups: ≤ 50 years old patients and > 50 years old patients the serotype 3 was significantly more frequent in older adults (Additional file 1: Table S3). The rest of the results regarding serotype distribution are presented in Tables 3, 4 and Fig. 2.

**Table 4:** Serotype distribution of *Streptococcus pneumoniae* grouped by age (n (%))

Serotype distribution	Age range	Age range	Age range	Age range	P value
	(18–35) years n = 38	(36–50) years n = 62	(51–64) years n = 74	(≥ 65) years n = 136	
19A	4 (10.53)	12 (19.35)	11 (14.86)	15 (11.03)	0.40
3	0 (0)	7 (11.29)	10 (13.51)	22 (16.18)	0.06
14	5 (13.16)	3 (4.84)	4 (5.41)	6 (4.41)	0.22
15A	2 (5.26)	4 (6.45)	3 (4.05)	7 (5.15)	0.94
23A	1 (2.63)	1 (1.61)	7 (9.46)	7 (5.15)	0.18
6C	1 (2.63)	2 (3.23)	4 (5.41)	9 (6.62)	0.66
11A	3 (7.89)	1 (1.61)	2 (2.7)	8 (5.88)	0.33
9N	3 (7.89)	1 (1.61)	2 (2.7)	7 (5.15)	0.38
19F	1 (2.63)	1 (1.61)	1 (1.35)	5 (3.68)	0.72
6A	0 (0)	1 (1.61)	4 (5.41)	3 (2.21)	0.30
23B	0 (0)	4 (6.45)	1 (1.35)	1 (0.74)	0.03
15B	2 (5.26)	0 (0)	2 (2.7)	3 (2.21)	0.38
22F	1 (2.63)	2 (3.23)	2 (2.7)	2 (1.47)	0.86
8	2 (5.26)	2 (3.23)	0 (0)	3 (2.21)	0.31
1	0 (0)	2 (3.23)	2 (2.7)	2 (1.47)	0.64
10A	0 (0)	1 (1.61)	1 (1.35)	4 (2.94)	0.65
23F	1 (2.63)	4 (6.45)	0 (0)	1 (0.74)	**0.02**
7F	1 (2.63)	0 (0)	1 (1.35)	4 (2.94)	0.53
9V	0 (0)	3 (4.84)	2 (2.7)	1 (0.74)	0.19
16F	1 (2.63)	0 (0)	0 (0)	3 (2.21)	0.36
34	1 (2.63)	2 (3.23)	1 (1.35)	1 (0.74)	0.58
6B	0 (0)	2 (3.23)	0 (0)	3 (2.21)	0.36
13	1 (2.63)	0 (0)	2 (2.7)	1 (0.74)	0.41
18C	0 (0)	0 (0)	2 (2.7)	2 (1.47)	0.47
12B	0 (0)	1 (1.61)	2 (2.7)	0 (0)	0.45
20	0 (0)	1 (1.61)	0 (0)	2 (1.47)	0.63
28A	1 (2.63)	0 (0)	0 (0)	2 (1.47)	0.42
35B	0 (0)	0 (0)	0 (0)	3 (2.21)	0.27
35F	0 (0)	1 (1.61)	1 (1.35)	1 (0.74)	0.84
37	2 (5.26)	1 (1.61)	0 (0)	0 (0)	**0.02**
7C	0 (0)	0 (0)	1 (1.35)	2 (1.47)	0.69
12F	1 (2.63)	0 (0)	1 (1.35)	0 (0)	0.24
25F	1 (2.63)	1 (1.61)	0 (0)	0 (0)	0.20
31	0 (0)	0 (0)	1 (1.35)	1 (0.74)	0.74
35A	0 (0)	0 (0)	0 (0)	2 (1.47)	0.46
4	2 (5.26)	0 (0)	0 (0)	0 (0)	< 0.01
11D	0 (0)	0 (0)	1 (1.35)	0 (0)	0.36
15C	0 (0)	1 (1.61)	0 (0)	0 (0)	0.26
16	0 (0)	0 (0)	1 (1.35)	0 (0)	0.36
17F	1 (2.63)	0 (0)	0 (0)	0 (0)	0.06
18A	0 (0)	1 (1.61)	1 (1.35)	0 (0)	0.45
19C	0 (0)	0 (0)	0 (0)	1 (0.74)	0.73
24F	0 (0)	0 (0)	0 (0)	1 (0.74)	0.73
29	0 (0)	0 (0)	1 (1.35)	0 (0)	0.36
6D	0 (0)	0 (0)	0 (0)	1 (0.74)	0.73

Bold values represent the p values that are < 0.05

N, number; %, percentage

Primary and secondary outcomes were also assessed and presented stratified by the clinical diagnosis. All-cause mortality was 30.3% (94/310) in the whole cohort, 31.1% (58/186) in patients with CAP, 27.6% (16/58) in patients with meningitis and 30.3% (20/66) in patients with another diagnosis. The median (IQR) length of ICU stay was 2 days (0–7), 1.5 days (0–8) in CAP patients, 3 days (0–8) in meningitis patients and 1.5 (0–4.25) days in patients with another diagnosis. More than half of the patients were admitted to the ICU (52.6% [163/186]), with meningitis being the most frequent diagnosis on ICU admission. The overall population received a median of 10 (3–14) days of antibiotic treatment. Patients diagnosed with meningitis required longer duration of antibiotic treatment (14 [4–18] P = 0.02). Overall, 43.5% (81/186) of the patients with CAP, 62.1% (36/58) of those with meningitis and 36.4% (24/66) of those with another diagnosis required invasive mechanical ventilation. The need for mechanical ventilation in patients with meningitis was significantly higher (p = 0.01) (Table 5).

**Table 5:** Primary and secondary outcomes of patients hospitalized due to invasive pneumococcal disease (IPD)

Primary and Secondary Outcomes	All patients (n = 310)	Pneumonia (n = 186)	Meningitis (n = 58)	Others (n = 66)	P value
Overall mortality, n (%)	94 (30.3)	58 (31.1)	16 (27.6)	20 (30.3)	0.873
Mortality in the ICU, n (%)	64 (20.6)	38 (20.4)	10 (17.2)	16 (24.2)	0.626
Need for invasive mechanical ventilation, n (%)	141 (45.5)	81 (43.5)	36 (62.1)	24 (36.4)	0.012
Need for non-invasive mechanical ventilation, n (%)	16 (5.1)	14 (7.5)	1 (1.7)	1 (1.5)	0.131
ICU Admission, n (%)	163 (52.6)	97 (52.2)	37 (63.8)	29 (43.9)	0.086
Length of ICU stay, median (IQR)	2 (0–7)	1.5 (0–8)	3 (0–8)	1.5 (0–4.75)	0.598
Length of hospital stay, median (IQR)	12 (4–21)	10 (4–21)	15 (3.75–25)	9 (3–22.25)	0.061
Time to clinical stability, median (IQR)	3 (0–10)	3 (0–10)	4 (0–10)	3.5 (0–7)	0.903
Duration of antibiotic treatment, median (IQR)	10 (3–14)	10 (3–14)	14 (4–18)	7.5 (2–14)	0.029

## Discussion

Results from this study indicate that adult patients hospitalized due to IPD in Bogotá, Colombia are more frequently infected by pneumococcal serotypes included in the conjugate vaccine PCV13 and the unconjugated vaccine PPV23. Most of the serotypes associated with IPD were isolated from blood cultures, the most prevalent being serotype 19A, and 3 which are included in PCV13 and PPV23 but not on PCV10. We found that CAP was the primary clinical syndrome causing IPD, followed by meningitis. Finally, many of the patients had underlying comorbidities, such as hypertension and smoking; or were on chronic medications, which are risks for disease severity, and worse outcomes on IPD patients [9, 32].

There has been a significant decrease in the overall incidence of IPD in the United States and the United Kingdom since the introduction and widespread use of the 7-valent pneumococcal conjugate vaccine (PCV7) in children back in 2000, and the 13-valent pneumococcal conjugate vaccine (PVC13) in 2010 [33]. In children, this reduction was due to declines in the incidence of IPD in the vaccinated population, while in adults was presumably secondary to indirect effects on pneumococcal transmission via herd protection [34, 35]. However, simultaneous increases in the proportion of cases of IPD due to pneumococcal serotypes not included in the PCV7 or PCV13 vaccines have been observed [27, 36, 37].

Similarly, in Colombia, several studies have investigated serotype distribution before the introduction of PCV10 mass vaccination in young children at the end of 2011 [38]. Importantly, this is the only currently conjugated vaccine included in the Colombian obligatory national immunization program. Parra et al. [5] concluded that serotype 14 was the most prevalent pneumococcal serotype in children and serotype 3 in older adults between 2005 and 2010. Later, in 2019 Camacho Moreno et al*.* [39] found that serotype 19A was the leading cause of IPD in Colombian infants, being pneumonia its principal clinical presentation, followed by bacteremia and meningitis that is consistent with our results that evidenced that pneumonia and meningitis are the main clinical presentation in adults. Likewise, these findings provided clinical data regarding serotype distribution in infants and evidenced the serotype replacement that followed the massive undertake of PCV10 in pediatric population in Colombia. There has not been a prior study to document similar findings in an adult population in Colombia. Our study presents for the first time, that as described in children, strains 19A and 3 are the main cause of IPD in Colombian adults. Interestingly, serotype 3 did not infect young adults (18–35 years old) but was the leading cause of IPD in patients ≥ 65 years old. Those findings highlight the importance of prioritizing vaccination programs in adults as pneumococcal disease continues to be a highly prevalent infection, and the most common serotypes causing IPD in Colombia are included in currently available adult pneumococcal vaccines.

Our results show that the most prevalent pneumococcal serotypes are not included in the PCV10, which is part of the obligatory immunization program in Colombia for children < 2 years old. Paradoxically and despite the Colombian guideline recommendations for the use of PCV13 and PPV23 in adult population, serotypes 19A and 3 were the main cause of IPD in our cohort. This phenomenon may be explained by the low vaccine rate in adults (Table 1). Additionally, PCV 13 has been shown to have low immunogenicity for serotype 3 and 19A, which might also contribute to the high prevalence of these serotypes [34, 35]. Yet, this requires further study since vaccination uptakes is still low in Colombia. Taking these potential explanations into account, it is important to promote public health programs for adult vaccination with PCV13 and PPV23 in adults and continuously monitor the serotype frequency to decide which pneumococcal vaccines to use and which should be targeted population.

Our study is subjected to limitations and strengths that are important to acknowledge. First, we analyzed a relatively small cohort of patients; however, it is a multicentre study that provides a valuable number of cases. Moreover, the data presented in this manuscript might serve as starting point to carry out a bigger study that can truly represent Colombian IPD. Furthermore, being a study in Colombia alone, it may not be replicable to other countries, although the major findings were similar to that of those previously published international studies [22, 40–42]. Moreover, as it is a retrospective study, some information was missing from medical records, leading to an abnormal data distribution of some of the physiological variables and exclusion of several patients. Additionally, due to the characteristics of our health system, there is several discrepancies about the number of days patients are treated for IPD; thus, readers should only extrapolate these results to countries with similar health care systems. We did not assess ICU mortality rate because not all patients were admitted to a hospital with ICU capabilities. Another limitation is that we did not follow the patients over time; thus, we cannot analyze long-term outcomes. Nevertheless, the results appear to reflect the overall trend of changes in the *Spn* serotype distribution in Colombia, with the inclusion of around 50% of isolates during a period of 8 years from the capital city. Importantly, we studied only adult patients, which is an essential strength of our study because even though children develop IPD more frequently than adults, the older population has a higher mortality rate. The surveillance isolation program is critical to identify serotype replacement and to monitor changes in serotype distribution that can also help decide with serotypes to include in newer vaccines. It is also important to mention that this study did not consider IPD incidence rate in adults but focus on the serotype distribution. Despite the limitations, our study shows that *Spn* continues to cause severe IPD in Colombian adults and also an impact in the health system resource use as evidenced by the high case fatality rate, the long length of stay and use of ICU. Continuous monitoring of serotypes and pneumococcal disease is necessary for a better understanding of the pneumococcal burden trends and epidemiology.

In conclusion, this study provides information on the current circulating pneumococcal serotypes in adults hospitalized due to IPD in Bogota, Colombia. Pneumococcal pneumonia continues to be the most prevalent cause of IPD in this population. Comorbidities and smoking are commonly found in these patients therefore should be carefully observed to achieve early diagnosis and treatment. Further research is needed to continue surveillance for potential switching to (PCV13) or to higher valent pediatric and adult conjugate vaccines (e.g., PCV15 or PCV20). Likewise, there is a necessity in the Colombian adult population to promote pneumococcal vaccination as recommended by the national guidelines. More extensive prospective studies are necessary to assess future changes in the epidemiology and to make the best decisions regarding immunization strategies.

## Supplementary Information

**Additional file 1: Table S1.** Posible associations between Spn srotypes and patients demographic characteristics/outcomes. **Table S2.** Hospitalization characteristics and medical interventions in patients hospitalized due to Invasive Pneumococcal Disease (IPD). **Table S3.** Serotype distribution of *Streptococcus pneumoniae* grouped by age: 18–50 years old Vs > 50 years old (n (%)). **Fig. S1.** Study’s flowchart.

## Data Availability

The datasets used and/or analyzed during the current study available from the corresponding author on reasonable request.

## References

[1] Centers for Disease C. Invasive Pneumococcal Disease—case definition. 2017 (18/05/2021). https://wwwn.cdc.gov/nndss/conditions/invasive-pneumococcal-disease/case-definition/2017/.

[2] Randle E, Ninis N, Inwald D (2011). Invasive pneumococcal disease. Arch Dis Child Educ Pract Ed.

[3] Feldman C, Anderson R (2014). Recent advances in our understanding of *Streptococcus pneumoniae* infection. F1000prime Rep..

[4] Centers for Disease C. Report findings—*Streptococcus pneumoniae* 2018 (18/05/2021). https://www.cdc.gov/abcs/reports-findings/survreports/spneu18.html.

[5] Parra EL, Ramos V, Sanabria O, Moreno J (2014). Serotype and genotype distribution among invasive *Streptococcus pneumoniae* isolates in Colombia, 2005–2010. PLoS ONE.

[6] Severiche-Bueno DF, Severiche-Bueno DF, Bastidas A, Caceres EL, Silva E, Lozada J (2021). Burden of invasive pneumococcal disease (IPD) over a 10-year period in Bogota, Colombia. Int J Infect Dis.

[7] Bardach AE, Rey-Ares L, Calderon Cahua M, Ciapponi A, Cafferata ML, Cormick G (2017). Burden of culture-confirmed pediatric *Pneumococcal pneumoni*a in Latin America and the Caribbean: a systematic review and meta-analysis. Value Health Reg Issues..

[8] Jain S, Self WH, Wunderink RG, Fakhran S, Balk R, Bramley AM (2015). Community-acquired pneumonia requiring hospitalization among U.S. adults. N Engl J Med.

[9] Aliberti S, Cook GS, Babu BL, Reyes LF, Rodriguez AH, Sanz F (2019). International prevalence and risk factors evaluation for drug-resistant *Streptococcus pneumoniae* pneumonia. J Infect.

[10] Aliberti S, Reyes LF, Faverio P, Sotgiu G, Dore S, Rodriguez AH (2016). Global initiative for meticillin-resistant *Staphylococcus aureus* pneumonia (GLIMP): an international, observational cohort study. Lancet Infect Dis.

[11] Bonten MJ, Huijts SM, Bolkenbaas M, Webber C, Patterson S, Gault S (2015). Polysaccharide conjugate vaccine against *Pneumococcal pneumonia* in adults. N Engl J Med.

[12] Wunderink RG, Feldman C (2020). Community-acquired pneumonia: a global perspective. Semin Respir Crit Care Med..

[13] Van der Poll T, Opal SM (2009). Pathogenesis, treatment, and prevention of pneumococcal pneumonia. Lancet.

[14] Diao WQ, Shen N, Yu PX, Liu BB, He B (2016). Efficacy of 23-valent pneumococcal polysaccharide vaccine in preventing community-acquired pneumonia among immunocompetent adults: a systematic review and meta-analysis of randomized trials. Vaccine.

[15] Mirsaeidi M, Ebrahimi G, Allen MB, Aliberti S (2014). Pneumococcal vaccine and patients with pulmonary diseases. Am J Med.

[16] Feldman C, Anderson R (2020). Recent advances in the epidemiology and prevention of *Streptococcus pneumoniae i*nfections. F1000Res.

[17] Bryant KA, Block SL, Baker SA, Gruber WC, Scott DA, Group PCVIS (2010). Safety and immunogenicity of a 13-valent pneumococcal conjugate vaccine. Pediatrics.

[18] Pilishvili T, Bennett NM (2015). Pneumococcal disease prevention among adults: strategies for the use of pneumococcal vaccines. Am J Prev Med.

[19] Yeh SH, Gurtman A, Hurley DC, Block SL, Schwartz RH, Patterson S (2010). Immunogenicity and safety of 13-valent pneumococcal conjugate vaccine in infants and toddlers. Pediatrics.

[20] Matanock A, Lee G, Gierke R, Kobayashi M, Leidner A, Pilishvili T (2019). Use of 13-valent pneumococcal conjugate vaccine and 23-valent pneumococcal polysaccharide vaccine among adults aged >/=65 years: updated recommendations of the Advisory Committee on immunization practices. MMWR Morb Mortal Wkly Rep.

[21] Hicks LA, Harrison LH, Flannery B, Hadler JL, Schaffner W, Craig AS (2007). Incidence of pneumococcal disease due to non-pneumococcal conjugate vaccine (PCV7) serotypes in the United States during the era of widespread PCV7 vaccination, 1998–2004. J Infect Dis.

[22] Agudelo CI, Castaneda-Orjuela C, Brandileone MCC, Echaniz-Aviles G, Almeida SCG, Carnalla-Barajas MN (2021). The direct effect of pneumococcal conjugate vaccines on invasive pneumococcal disease in children in the Latin American and Caribbean region (SIREVA 2006–17): a multicentre, retrospective observational study. Lancet Infect Dis.

[23] Castaneda-Orjuela C, De la Hoz-Restrepo F (2018). How cost effective is switching universal vaccination from PCV10 to PCV13? A case study from a developing country. Vaccine.

[24] Duarte C, Sanabria O, Moreno J (2013). Molecular characterization of *Streptococcus pneumoniae* serotype 1 invasive isolates in Colombia. Rev Panam Salud Publica.

[25] Harboe ZB, Thomsen RW, Riis A, Valentiner-Branth P, Christensen JJ, Lambertsen L (2009). Pneumococcal serotypes and mortality following invasive pneumococcal disease: a population-based cohort study. PLoS Med.

[26] Habib M, Porter BD, Satzke C (2014). Capsular serotyping of *Streptococcus pneumoniae* using the Quellung reaction. J Vis Exp.

[27] Yildirim I, Shea KM, Pelton SI (2015). Pneumococcal disease in the era of pneumococcal conjugate vaccine. Infect Dis Clin North Am.

[28] Metlay JP, Waterer GW, Long AC, Anzueto A, Brozek J, Crothers K (2019). Diagnosis and treatment of adults with community-acquired pneumonia. An official clinical practice guideline of the American Thoracic Society and Infectious Diseases Society of America. Am J Respir Crit Care Med.

[29] Tunkel AR, Hartman BJ, Kaplan SL, Kaufman BA, Roos KL, Scheld WM (2004). Practice guidelines for the management of bacterial meningitis. Clin Infect Dis.

[30] Vincent JL, de Mendonca A, Cantraine F, Moreno R, Takala J, Suter PM (1998). Use of the SOFA score to assess the incidence of organ dysfunction/failure in intensive care units: results of a multicenter, prospective study. Working group on "sepsis-related problems" of the European Society of Intensive Care Medicine. Crit Care Med.

[31] Headley J, Theriault R, Smith TL. Independent validation of APACHE II severity of illness score for predicting mortality in patients with breast cancer admitted to the intensive care unit. Cancer. 1992;70(2):497–503. https://doi.org/10.1002/1097-0142(19920715)70:2<497::aid-cncr2820700220>3.0.co;2-h. (Epub 1992/07/15). 10.1002/1097-0142(19920715)70:2<497::aid-cncr2820700220>3.0.co;2-h 1617599

[32] Torres A, Blasi F, Dartois N, Akova M (2015). Which individuals are at increased risk of pneumococcal disease and why? Impact of COPD, asthma, smoking, diabetes, and/or chronic heart disease on community-acquired pneumonia and invasive pneumococcal disease. Thorax.

[33] Weinberger DM, Warren JL, Dalby T, Shapiro ED, Valentiner-Branth P, Slotved HC (2019). Differences in the impact of pneumococcal serotype replacement in individuals with and without underlying medical conditions. Clin Infect Dis.

[34] Weinberger DM, Malley R, Lipsitch M (2011). Serotype replacement in disease after pneumococcal vaccination. Lancet.

[35] Centers for Disease C, Prevention. Direct and indirect effects of routine vaccination of children with 7-valent pneumococcal conjugate vaccine on incidence of invasive pneumococcal disease--United States, 1998–2003. MMWR Morb Mortal Wkly Rep. 2005;54(36):893–7 (Epub 2005/09/16). 16163262

[36] Lexau CA, Lynfield R, Danila R, Pilishvili T, Facklam R, Farley MM (2005). Changing epidemiology of invasive pneumococcal disease among older adults in the era of pediatric pneumococcal conjugate vaccine. JAMA.

[37] Albrich WC, Baughman W, Schmotzer B, Farley MM (2007). Changing characteristics of invasive pneumococcal disease in Metropolitan Atlanta, Georgia, after introduction of a 7-valent pneumococcal conjugate vaccine. Clin Infect Dis.

[38] Caceres DC, Ortega-Barria E, Nieto J, DeAntonio R (2018). Pneumococcal meningitis trends after pneumococcal conjugate vaccine introduction in Colombia: an interrupted time-series analysis. Hum Vaccin Immunother.

[39] Moreno CG, Imbachi LF, Leal AL, Moreno VM, Patino JA, Gutierrez IF (2020). Emergence of *Streptococcus pneumoniae* serotype 19A (Spn19A) in the pediatric population in Bogota, Colombia as the main cause of invasive pneumococcal disease after the introduction of PCV10. Hum Vaccin Immunother.

[40] Galanis I, Lindstrand A, Darenberg J, Browall S, Nannapaneni P, Sjostrom K (2016). Effects of PCV7 and PCV13 on invasive pneumococcal disease and carriage in Stockholm, Sweden. Eur Respir J.

[41] Jiang H, Huai Y, Chen H, Uyeki TM, Chen M, Guan X (2018). Invasive *Streptococcus pneumoniae* infection among hospitalized patients in Jingzhou city, China, 2010–2012. PLoS ONE.

[42] Demczuk WHB, Martin I, Desai S, Griffith A, Caron-Poulin L, Lefebvre B (2018). Serotype distribution of invasive *Streptococcus pneumoniae* in adults 65years of age and over after the introduction of childhood 13-valent pneumococcal conjugate vaccination programs in Canada, 2010–2016. Vaccine.

