# Supplementary material for: Invasive pneumococcal disease burden in hospitalized adults in Bogota, Colombia
Source: BMC Infect Dis. 2021 Oct 12;21:1059. doi: 10.1186/s12879-021-06769-2 (PMC8507327; doi:10.1186/s12879-021-06769-2)

**S1 Table. Posible associations between Spn srotypes and patients demographic characteristics/outcomes.**

| *spn* serotype | **Smoking** | **Alcoholism** | **COPD** | **DM** | **HT** | **HF** | **CRI** | **HD** | **OB** | **HIV** | **CAD** | **AF** | **AID** | **CA** | **EP** | **MACE** |  |  |
| --- | --- | --- | --- | --- | --- | --- | --- | --- | --- | --- | --- | --- | --- | --- | --- | --- | --- | --- |
| 19A |  |  |  |  |  |  |  |  |  |  |  |  |  |  |  |  |  |  |
| 3 | 0,028 |  |  |  |  | 0,011 |  |  |  |  |  |  |  |  |  | 0,039 |  |  |
| 14 |  |  |  |  |  |  |  |  |  |  |  |  |  |  |  |  |  |  |
| 15A |  |  |  |  |  |  |  |  |  |  |  |  |  |  |  |  |  |  |
| 23A |  |  |  |  |  |  |  |  | 0,012 |  |  |  |  |  |  |  |  |  |
| 6C |  |  |  |  | 0,001 | 0,025 |  | 0,017 | 0,012 |  | 0,014 |  |  |  |  |  |  |  |
| 11A |  |  |  |  |  |  | 0,043 |  |  |  |  |  |  |  |  |  |  |  |
| 9N |  |  |  |  |  | 0,05 |  |  |  |  |  |  |  |  |  | 0,042 |  |  |
| 19F |  |  |  |  |  |  |  |  |  |  |  |  |  |  |  |  |  |  |
| 6A |  | 0,001 |  |  |  |  |  | 0,014 |  |  |  |  |  |  |  |  |  |  |
| 23B |  |  |  |  | 0,034 |  |  |  |  |  |  |  |  |  |  |  |  |  |
| 15B |  |  |  |  |  |  |  |  |  |  |  |  |  |  |  |  |  |  |
| 22F |  |  |  |  |  |  |  |  |  |  |  |  |  |  |  |  |  |  |
| 8 |  |  | 0,028 |  |  |  |  |  |  |  |  |  |  |  |  |  |  |  |
| 1 |  |  |  |  |  |  |  |  |  |  |  |  |  |  |  |  |  |  |
| 10A |  |  |  |  |  |  |  |  |  |  |  |  | 0,004 |  |  |  |  |  |
| 23F |  |  |  |  |  |  |  |  |  | 0,002 |  | 0,04 |  |  |  |  |  |  |
| 7F | 0,038 | 0,004 |  |  |  |  |  |  |  |  |  |  |  |  |  |  |  |  |
| 9V |  | 0,004 |  |  | 0,034 |  |  |  |  |  |  |  |  |  |  |  |  |  |
| 16F |  |  |  |  |  |  | 0,016 |  |  |  |  |  |  |  |  |  |  |  |
| 34 |  |  |  |  |  |  |  |  |  |  |  |  |  |  | 0,001 |  |  |  |
| 6B |  |  |  |  |  |  |  |  |  |  | 0,01 |  |  |  |  |  |  |  |
| 13 |  |  |  |  |  |  |  |  |  |  |  |  |  |  |  |  |  |  |
| 18C |  |  |  |  |  |  |  |  |  |  |  |  |  |  |  |  |  |  |
| 12B |  |  |  |  |  |  |  |  |  |  |  |  |  |  |  |  |  |  |
| 20 |  |  |  |  |  |  |  | 0,033 |  |  |  |  |  |  |  |  |  |  |
| 28A |  |  |  |  |  |  |  | 0,033 |  |  |  |  | 0,004 |  |  |  |  |  |
| 35B |  |  |  |  |  |  |  |  |  |  |  |  |  | 0,003 |  |  |  |  |
| 35F |  |  |  |  |  |  |  |  |  |  |  |  |  |  |  |  |  |  |
| 37 |  |  |  |  |  |  |  |  | 0,027 |  |  |  | 0,004 |  |  |  |  |  |
| 7C |  |  |  |  |  |  |  | 0,033 |  |  |  |  |  |  |  |  |  |  |
| 12F | 0,025 |  |  |  |  |  |  |  |  |  |  |  |  |  |  |  |  |  |
| 25F |  |  |  |  |  |  |  |  |  |  |  |  |  |  |  |  |  |  |
| 31 |  |  |  |  |  |  |  |  |  |  |  |  |  |  |  |  |  |  |
| 35A |  |  |  |  |  |  |  |  |  |  |  |  |  |  |  |  |  |  |
| 4 |  |  |  |  |  |  |  |  |  |  |  |  |  |  |  |  |  |  |
| 11D |  |  |  |  |  |  |  |  |  |  |  |  |  |  |  |  |  |  |
| 15C |  |  |  |  |  |  |  |  |  |  |  |  |  |  |  |  |  |  |
| 16 |  |  |  |  |  |  |  |  |  |  |  |  |  |  |  |  |  |  |
| 17F |  | 0,007 |  |  |  |  |  |  |  |  |  |  |  |  |  |  |  |  |
| 18A |  |  |  |  |  |  |  |  |  |  | 0,033 |  |  |  |  |  |  |  |
| 19C |  |  | 0,014 |  |  |  |  |  |  |  |  |  | 0,007 |  |  |  |  |  |
| 24F |  |  |  | 0,022 |  | 0,009 |  |  |  |  | 0,001 |  |  |  |  |  |  |  |
| 29 |  |  |  |  |  |  |  |  |  |  |  |  | 0,007 |  |  |  |  |  |
| 6D |  |  |  |  |  |  |  |  |  |  |  |  |  | 0,007 |  |  |  |  |
| **COPD, chornic obstructivepulmonary disease; DM, diabetes mellitus; HT, Hypertension; HF, heart failure; CRF, Chronic renal failure; HD, hepatic disease; OB, Obesity; HIV, Human immunodeficiency virus; CAD, Coronary artery disease; AF, Atrial fibrilation; AID, Autoimmune disease; CA, Cancer;EP, Epilepsy; MACE, Major adverse cardiovascular events.** | | | | | | | | | | | | | | | | | |  |
|  |  |  |  |  |  |  |  |  |  |  |  |  |  |  |  |  |  |  |

**S2 Table. Hospitalization characteristics and medical interventions in patients hospitalized due to Invasive Pneumococcal Disease (IPD).**

| **Hospitalization characteristics and medical interventions** | **All patients**  **(n= 310)** | **Pneumonia (n= 186)** | **Meningitis**  **(n= 58)** | **Others**  **(n= 66)** |
| --- | --- | --- | --- | --- |
| **Vasopressor Therapy** | | | | |
| Noradrenaline | 131 (42.3) | 82 (44.1) | 26 (44.8) | 23 (34.8) |
| Vasopressin | 50 (16.1) | 36 (19.4) | 7 (12.1) | 7 (10.6) |
| Adrenaline | 19 (6.1) | 9 (4.8) | 3 (5.2) | 7 (10.6) |
| Dopamine | 9 (-2.9) | 7 (3.8) | 2 (3.4) | 0 (0) |
| **Inotropic Therapy** | | | | |
| Levosimendan | 6 (1.9) | 4 (2.2) | 0 (0) | 2 (3) |
| Dobutamine | 25 (8.1) | 16 (8.6) | 4 (6.9) | 5 (7.6) |
| **Corticosteroid therapy** | | | | |
| Dexamethasone | 42 (13.5) | 9 (4.8) | 28 (48.3) | 5 (7.6) |
| Hydrocortisone | 52 (16.8) | 38 (20.4) | 6 (10.3) | 8 (12.1) |
| **Multimodal analgesia** | | | | |
| Midazolam | 133 (42.9) | 72 (38.7) | 39 (67.2) | 22 (33.3) |
| Fentanyl | 143 (46.1) | 78 (41.9) | 39 (67.2) | 26 (39.4) |
| Dexmedetomidine | 26 (8.4) | 13 (7) | 9 (15.5) | 4 (6.1) |
| Propofol | 28 (9) | 10 (5.4) | 12 (20.7) | 6 (9.1) |
| Remifentanil | 7 (2.3) | 2 (1.1) | 3 (5.2) | 2 (3) |
| **Antibiotic treatment** | | | | |
| Ceftriaxone | 81 (26.1) | 18 (9.7) | 51 (87.9) | 12 (18.2) |
| Piperacillin/tazobactam | 116 (37.4) | 86 (46.2) | 6 (10.3) | 24 (36.4) |
| Cefepime | 50 (16.1) | 33 (17.7) | 9 (15.5) | 8 (12.1) |
| Meropenem | 23 (7.4) | 14 (7.5) | 5 (8.6) | 4 (6.1) |
| Ertapenem | 1 (0.3) | 0 (0) | 0 (0) | 1 (1.5) |
| Polymyxin B | 1 (0.3) | 1 (0.5) | 0 (0) | 0 (0) |
| Colistin | 1 (0.3) | 0 (0) | 0 (0) | 1 (1.5) |
| Fosfomycin | 1 (0.3) | 0 (0) | 0 (0) | 1 (1.5) |
| Ciprofloxacin | 3 (1) | 1 (0.5) | 0 (0) | 2 (3) |
| Vancomicin | 126 (40.6) | 59 (31.7) | 49 (84.5) | 18 (27.3) |
| Linezolid | 18 (5.8) | 15 (8.1) | 1 (1.7) | 2 (3) |
| Daptomycin | 3 (1) | 3 (1.6) | 0 (0) | 0 (0) |
| Gentamicin | 2 (0.6) | 0 (0) | 1 (1.7) | 1 (1.5) |
| Rifampicin | 3 (1) | 3 (1.6) | 0 (0) | 0 (0) |
| Trimethoprim sulfamethoxazole | 11 (3.5) | 7 (3.8) | 2 (3.4) | 2 (3) |
| Ampicillin/sulbactam | 83 (26.8) | 63 (33.8) | 5 (8.6) | 15 (22.7) |
| Cefepime | 15 (4.8) | 10 (5.4) | 3 (5.2) | 2 (3) |
| Ampicillin | 22 (7.1) | 4 (2.2) | 14 (24.1) | 4 (6.1) |
| Clindamycin | 10 (3.2) | 5 (2.7) | 1 (1.7) | 4 (6.1) |
| Cefazolin | 8 (2.6) | 5 (2.7) | 0 (0) | 3 (4.5) |
| Cefuroxime | 1 (0.3) | 1 (0.5) | 0 (0) | 0 (0) |
| Clarithromycin | 81 (26.1) | 73 (39.2) | 1 (1.7) | 7 (10.6) |
| Oxacillin | 7 (2.3) | 3 (1.6) | 0(0) | 4 (6.1) |
| ***Streptococcus pneumoniae* Isolation** | | | | |
| Blood culture | 273 (88.1) | 172 (92.5) | 47 (81) | 54 (81.8) |
| Pleural fluid | 20 (6.5) | 19 (10.2) | 0 (0) | 1 (1.5) |
| Bronchoalveolar lavage | 19 (6.1) | 17 (9.1) | 0 (0) | 2 (3) |
| Cerebrospinal fluid | 44 (14.2) | 1 (0.5) | 41 (70.7) | 2 (3) |
| Ascitic fluid | 3 (1) | 0 (0) | 1 (1.7) | 2 (3) |
| **S1 Table**. describes all medical interventions required by the included cases in the study. The list of antibiotic treatment reflects all options given to the patients, including the ones given prior to the isolation of *Spn and once the Streptococcus pneumoniae was isolated.* | | | | |

**S3. Table. Serotype distribution of *Streptococcus pneumoniae* grouped by age: 18-50 years old Vs >50 years old (n (%))**

| Serotype distribution | Age range | Age range | P value |  |
| --- | --- | --- | --- | --- |
|  | (18-50) years n=100 | (>50) years n=210 (%) |  |  |
|  |  |  |  |  |
| 19A | 16 (16.0) | 26 (12.4) | 0.384 |  |
| 3 | 7 (7.0) | 32 (15.2) | **0.041** |  |
| 14 | 8 (8.0) | 10 (4.8) | 0.254 |  |
| 15A | 6 (6.0) | 10 (4.8) | 0.645 |  |
| 23A | 2 (2.0) | 14 (6.7) | 0.083 |  |
| 6C | 3 (3.0) | 13 (6.2) | 0.235 |  |
| 11A | 4 (4.0) | 10 (4.8) | 0.763 |  |
| 9N | 4 (4.0) | 9 (4.3) | 0.907 |  |
| 19F | 2 (2.0) | 6 (2.9) | 0.656 |  |
| 6A | 1 (1.0) | 7 (3.3) | 0.226 |  |
| 23B | 4 (4.0) | 2 (1.0) | 0.069 |  |
| 15B | 2 (2.0) | 5 (2.4) | 0.833 |  |
| 22F | 3 (3.0) | 4 (1.9) | 0.544 |  |
| 8 | 4 (4.0) | 3 (1.4) | 0.154 |  |
| 1 | 2 (2.0) | 4 (1.9) | 0.955 |  |
| 10A | 1 (1.0) | 5 (2.4) | 0.409 |  |
| 23F | 5 (5.0) | 1 (0.5) | **0.007** |  |
| 7F | 1 (1.0) | 5 (2.4) | 0.409 |  |
| 9V | 3 (3.0) | 3 (1.4) | 0.48 |  |
| 16F | 1 (1.0) | 3 (1.4) | 0.755 |  |
| 34 | 3 (3.0) | 2 (1.0) | 0.181 |  |
| 6B | 2 (2.0) | 3 (1.4) | 0.709 |  |
| 13 | 1 (1.0) | 3 (1.4) | 0.755 |  |
| 18C | 0 (0.0) | 4 (1.9) | 0.165 |  |
| 12B | 1 (1.0) | 1 (0.5) | 0.59 |  |
| 20 | 1 (1.0) | 2 (1.0) | 0.968 |  |
| 28A | 1 (1.0) | 2 (1.0) | 0.968 |  |
| 35B | 0 (0.0) | 3 (1.4) | 0.23 |  |
| 35F | 1 (1.0) | 2 (1.0) | 0.968 |  |
| 37 | 3 (3.0) | 0 (0.0) | **0.012** |  |
| 7C | 0 (0.0) | 3 (1.4) | 0.23 |  |
| 12F | 1 (1.0) | 1 (0.5) | 0.59 |  |
| 25F | 2 (2.0) | 0 (0.0) | **0.04** |  |
| 31 | 0 (0.0) | 2 (1.0) | 0.328 |  |
| 35A | 0 (0.0) | 2 (1.0) | 0.328 |  |
| 4 | 2 (2.0) | 0 (0) | **0.04** |  |
| 11D | 0 (0.0) | 1 (0.5) | 0.489 |  |
| 15C | 1 (1.0) | 0 (0.0) | 0.147 |  |
| 16 | 0 (0.0) | 1 (0.5) | 0.489 |  |
| 17F | 1 (1.0) | 0 (0.0) | 0.147 |  |
| 18A | 1 (1.0) | 1 (0.5) | 0.59 |  |
| 19C | 0 (0.0) | 1 (0.5) | 0.489 |  |
| 24F | 0 (0.0) | 1 (0.5) | 0.489 |  |
| 29 | 0 (0.0) | 1 (0.5) | 0.489 |  |
| 6D | 0 (0.0) | 1 (0.5) | 0.489 |  |

**S1 Fig. Study’s flowchart**


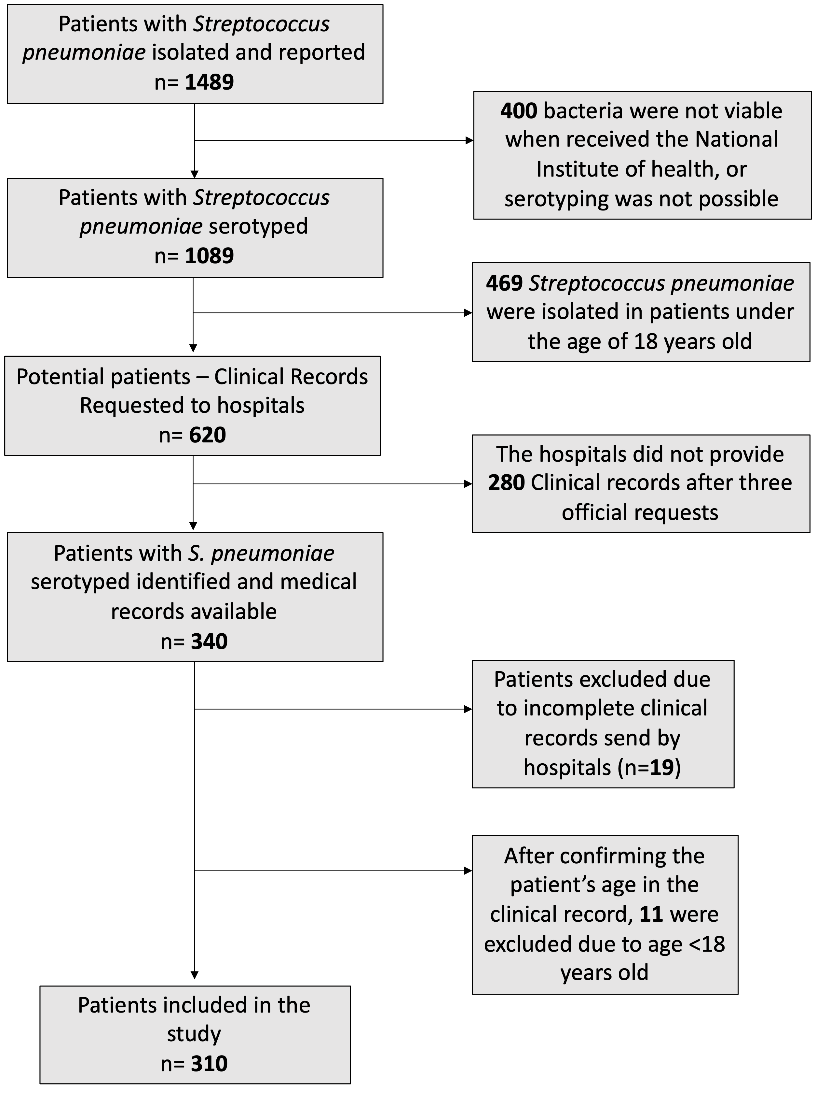

Supplement: Supplementary file 1 — Additional file 1: Table S1. Posible associations between Spn srotypes and patients demographic characteristics/outcomes. Table S2. Hospitalization characteristics and medical interventions in patients hospitalized due to Invasive Pneumococcal Disease (IPD). Table S3. Serotype distribution of Streptococcus pneumoniae grouped by age: 18–50 years old Vs > 50 years old (n (%)). Fig. S1. Study’s flowchart. [file 12879_2021_6769_MOESM1_ESM.docx]
